# Trop2 inhibition suppresses the proliferation and invasion of laryngeal carcinoma cells via the extracellular signal-regulated kinase/mitogen-activated protein kinase pathway

**DOI:** 10.3892/mmr.2015.3485

**Published:** 2015-03-13

**Authors:** XU-DONG WANG, QIANG WANG, XIAO-LIN CHEN, JIAN-FEI HUANG, YONG YIN, PENG DA, HAO WU

**Affiliations:** 1 Department of Laboratory Medicine, Affiliated Hospital of Nantong University, Nantong, Jiangsu 226000, P.R. China; 2 Department of Otolaryngology-Head and Neck Surgery, Affiliated Hospital of Nantong University, Nantong, Jiangsu 226000, P.R. China; 3 Department of Pathology, Affiliated Hospital of Nantong University, Nantong, Jiangsu 226000, P.R. China

**Keywords:** laryngeal carcinoma, Trop2, invasion, proliferation

## Abstract

The cell surface glycoprotein Trop2 is overexpressed in various types of epithelial cancer. Laryngeal carcinoma is one of the most common types of head and neck cancer and in a previous study, the expression of Trop2 in laryngeal squamous cell carcinoma (LSCC) was identified as an independent prognostic factor. However, the biological significance of Trop2 in LSCC development remains unclear. In the current study, Trop2 protein expression in fresh LSCC tissue and paracancerous tissue was investigated using western blotting. Trop2 in the Hep2 laryngeal cell line was subsequently suppressed by transfection with small interfering RNA (siRNA). The effects of knockdown of Trop2 on cell viability, migration, invasiveness and ERK/MAPK pathway activity were investigated in the current study. The expression of Trop2 in fresh LSCC tissue was demonstrated to be significantly greater than that in paracancerous tissue. Trop2 expression was also identified to be required for proliferation, migration and invasiveness of Hep2 laryngeal carcinoma cells, as all were blocked by siRNA-mediated Trop2 inhibition. Notably, the ERK/MAPK signaling pathway and cell cycle factor, cyclin D1, were identified to be suppressed following the knockdown of Trop2 in Hep2 cells. These observations suggest that Trop2 serves an oncogenic role in LSCC and has potential as a therapeutic target.

## Introduction

Laryngeal carcinoma is one of the most common types of head and neck cancer. Greater than 1.5 million individuals are diagnosed with head and neck squamous cell carcinoma annually worldwide, with ~25% represented by patients with laryngeal squamous cell carcinoma (LSCC) (1). Although progress has been made in the diagnosis and treatment of laryngeal carcinoma, significant improvements in survival remain to be achieved (2,3).

The Trop2 gene (also termed TACSTD2) is located on 1p32. It encodes for a single-pass transmembrane protein of 35.7 kDa, which contains a conserved motif involved in Trop2-mediated signaling (4,5). A previous study demonstrated that a phosphatidylinositol 4,5-bis phosphate-binding sequence is present in this motif (6). A conserved serine residue within this sequence is phosphorylated by protein kinase C (PKC) (6). Thus, PKC and mitogen-activated protein kinases (MAPKs), including extracellular signal-regulated kinase 1/2 (ERK1/2), may be associated with Trop2-mediated tumor cell activity (7). Trop2 is involved in the regulation of cell adhesion and its overexpression has been observed in a variety of epithelial cells, whereas in healthy human somatic cells and tissues, expression is either low or absent (8). It has been demonstrated that elevated expression of Trop2 in pancreatic, stomach, oral and cervical cancer is correlated with poor survival (9–12). In a previous study, it was demonstrated that the expression of Trop2 in laryngeal carcinoma is an independent prognostic factor (13). However, the biological significance of Trop2 in the development of LSCC remains to be fully elucidated.

In the present study, the role of Trop2 in laryngeal carcinoma was investigated. In order to establish this role, Trop2 expression was suppressed in the Hep2 human laryngeal carcinoma cell line using small interfering RNA (siRNA), and the effects of its knockdown on proliferation, migration and invasiveness were examined. The interaction between Trop2 and the ERK/MAPK signaling pathway were also investigated.

## Materials and methods

### Clinical samples

A total of four paired fresh laryngeal carcinoma tissues and adjacent non-cancerous tissues were collected from The Head and Neck Department of The Affiliated Hospital of Nantong University (AHNU, Nantong, China). The paraffin-embedded laryngeal carcinoma tissues were collected from the Department of Pathology of the AHNU. The current study was approved by the Medical Ethics Committee of the AHNU and samples were collected with informed patient consent.

### Cell culture

The Hep2 human laryngeal carcinoma cell line was purchased from the Type Culture Collection of the Chinese Academy of Sciences (Shanghai, China) and maintained in RPMI-1640 (Gibco Life Technologies, Grand Island, NY, USA) with 10% fetal bovine serum (FBS; Hangzhou Sijiqing Biological Engineering Materials Co., Ltd., Hangzhou, China), 100 U/ml penicillin and 100 mg/ml streptomycin (Gibco Life Technologies) at 37°C in a humidified atmosphere containing 5% CO_2_.

### siRNA transfection

Hep2 cells in the logarithmic growth phase were harvested and sub-cultured into 6-well plates. At 70–80% confluence, cells were transfected with Trop2 siRNAs (Table I; Guangzhou Ribobio Co., Ltd., Guangzhou, China) at 100 nmol using Lipofectamine 2000 (Invitrogen Life Technologies, Carlsbad, CA, USA). A non-targeting siRNA was used as a negative control (NC; Guangzhou Ribobio Co., Ltd.). After 24 h, fluorescence microscopy (BX51; Olympus Corporation, Tokyo, Japan) was used to examine transfection efficiency. Reverse transcription-quantitative polymerase chain reaction (RT-qPCR) was used to examine Trop2 mRNA expression profiles of the transfected cells, and the siRNA that induced the maximal suppression was selected for subsequent analysis.

### RT-qPCR

Total RNA was extracted from cells using TRIzol reagent (Invitrogen Life Technologies). First-strand complementary DNA synthesis was then performed using the Reverse Transcription System kit (Thermo Fisher Scientific, Pittsburgh, PA, USA) according to the manufacturer’s instructions. RT-qPCR was performed using the SYBR Green kit (Thermo Fisher Scientific) to examine Trop2 mRNA levels, in addition to those of GAPDH, which served as the endogenous control for normalization. The relative expression levels of Trop2 mRNA were calculated using the comparative 2^−ΔΔCt^ method. The qPCR was conducted on an Applied Biosystems 7500 Real-Time PCR system (Applied Biosystems, Foster City, CA, USA). The following RT-qPCR conditions were used: 2 min at 94°C, followed by 40 cycles of 15 sec at 94°C, 25 sec at 58°C and 30 sec at 72°C. Each experiment was performed three times in duplicate. Primer sequences are presented in Table II.

### Western blot analysis

Whole-cell lysates were prepared using radioimmunoprecipitation assay buffer with protease inhibitors (Thermo Fisher Scientific, Beijing, China) and protein concentrations were quantitated using the Bradford method (Bio-Rad Laboratories, Inc., Hercules, CA, USA). Subsequently, proteins were separated using 7% SDS-PAGE and blotted using standard procedures. Visualization of the specific proteins on polyvinylidene fluoride membranes (Bio-Rad Laboratories, Inc.) was accomplished with an enhanced chemiluminescence reaction (Pierce Biotechnology, Inc., Rockford, IL, USA), followed by scanning using the Odyssey Infrared Imaging system (Li-Cor Biosciences, Lincoln, NE, USA) and analysis using Quantity One 1-D Analysis version 4.62 software (Bio-Rad Laboratories, Inc.). Relative target protein expression was determined using the formula: Gray scale value of target protein/gray scale value of β-actin (Actin). The goat anti-human polyclonal antibody against Trop2 (1:500; cat. no. AF650) and horseradish peroxidase (HRP)-conjugated chicken anti-goat immunoglobulin (Ig)G antibody (1:2,000; cat. no. HAF019) were purchased from R&D Systems, Inc. (Minneapolis, MN, USA). The following anti-human antibodies: Rabbit polyclonal anti-ERK 1/2 (1:750; cat. no. sc-292838), rabbit polyclonal phosphorylated-ERK 1/2 (1:500; cat. no. sc-23759-R), rabbit polyclonal anti-cyclin D1 (1:1,000; cat. no. sc-717), mouse monoclonal anti-p27 (1:1,000; cat. no. sc-1641), rabbit polyclonal anti-actin (1:1,000; cat. no. sc-7210), goat anti-rabbit IgG-HRP (1:2,500; cat. no. sc-2004) and goat anti-mouse IgG-HRP (1:2,500; cat. no. sc-2005) were purchased from Santa Cruz Biotechnology, Inc. (Santa Cruz, CA, USA). All primary antibodies were used at an incubation temperature of 4°C overnight and all secondary antibodies were used for 2 h at an ambient temperature.

### Immunohistochemical staining

Briefly, the sections were deparaffinized, rehydrated and subjected to antigen retrieval by boiling in 10 mM citrate buffer, pH 6.0 for 15 min, prior to blocking in 10% normal goat serum. The sections were then incubated with human TROP-2 Polyclonal Ab (cat. no. AF650; R&D Systems, Inc.; dilution 1:50) overnight at 4°C. Subsequently, the sections were stained using the Anti-Goat HRP-DAB Cell and Tissue Staining kit (cat. no. CTS008; R&D Systems, Inc.) and counterstained with hematoxylin. The images were captured using an Olympus BX-51 light microscope system (Olympus Corporation).

### Cell viability

Hep2 laryngeal carcinoma cells were transferred into 96-well plates at a density of 1×10^3^ cells/well in a volume of 200 *μ*l. Following cultivation for 12, 24, 36 and 48 h, 20 *μ*l MTT (5 g/l; Sigma-Aldrich, St. Louis, MO, USA) was added to each well. Following incubation for a further 4 h, the supernatant was discarded, 150 *μ*l dimethyl sulfoxide was added to each well and the resulting mixture was agitated under ambient conditions for 10 min, or until the newly formed crystals were dissolved completely. The absorbance of each well was then determined at 570 nm using a microplate spectrophotometer (Multiskan GO; Thermo Fisher Scientific, Beijing, China). Triplicates were prepared for each sample and each time point. The average values were used to prepare a growth curve.

### Cell invasion

Hep2 cells in the logarithmic growth phase were used to prepare a single cell suspension of ~3×10^5^ cells/ml. Transwell chambers (Costar Transwell; Corning Incorporated, Tewksbury, MA, USA) were sterilized for 2 h in an ultra-clean cabinet under ultraviolet radiation. Maintaining sterile conditions, the upper side of the 24-well Transwell chambers were coated with 50 *μ*l of 1 mg/ml Matrigel gum (Matrigel™; BD Biosciences, Franklin Lakes, NJ, USA). Following solidification at 4°C, the mixture was hydrated with serum-free RPMI 1640 medium for 30 min at 37°C to provoke its reor-ganization into a basement membrane-like structure over the microporous membrane. The single cell suspensions (100 *μ*l) were then transferred onto the upper chambers of the micro-porous membranes while 15% FBS-RPMI 1640 medium (500 *μ*l) was added to the lower chamber. Five replicates were analyzed for each group. The chamber was removed following cultivation at 37°C for 12, 24 and 36 h. The residual medium and cells of the upper chamber were removed carefully with a swab. The chamber was then dried under ambient conditions for 30 min, followed by staining with 0.1% crystal violet at 37°C for 30 min. The number of cells on the underside of the chamber was counted in six fields of view (magnification, ×50; Olympus BX51).

### Wound scratch assay

Hep2 cells in the logarithmic growth phase were transferred into six-well culture plates at a density of 5×10^5^ cells/well. Four parallel samples for each transfection group were prepared. Once the cells had reached 60–70% confluence, a straight-line scratch (2 mm in width) was created along the longitudinal axis in the center of each well using a 20-*μ*l pipette tip. Floating cells that were scraped off in the process were removed by washing the plates with phosphate-buffered saline three times. The remaining cells were placed in 1% FBS-RPMI 1640 medium (2 ml/well) for 24 h, then the medium was then replaced with 10% FBS-RPMI 1640 for continued cultivation. The scratch width was examined under the microscope (Olympus BX51) at 0, 24, 48 and 72 h later. Three independent experiments were performed in which all experimental and control groups were analyzed in triplicate.

### Statistical analysis

Statistical analyses were conducted using SPSS software, version 18.0 (SPSS, Inc., Chicago, IL, USA) and data were expressed as the mean ± standard error. Pairwise comparisons were analyzed using Student’s t-test. Multi-group differences were measured using a one way analysis of variance. P<0.05 was considered to indicate a statistically significant difference.

## Results

### Trop2 expression in LSCC tissues

**Figure 1:**
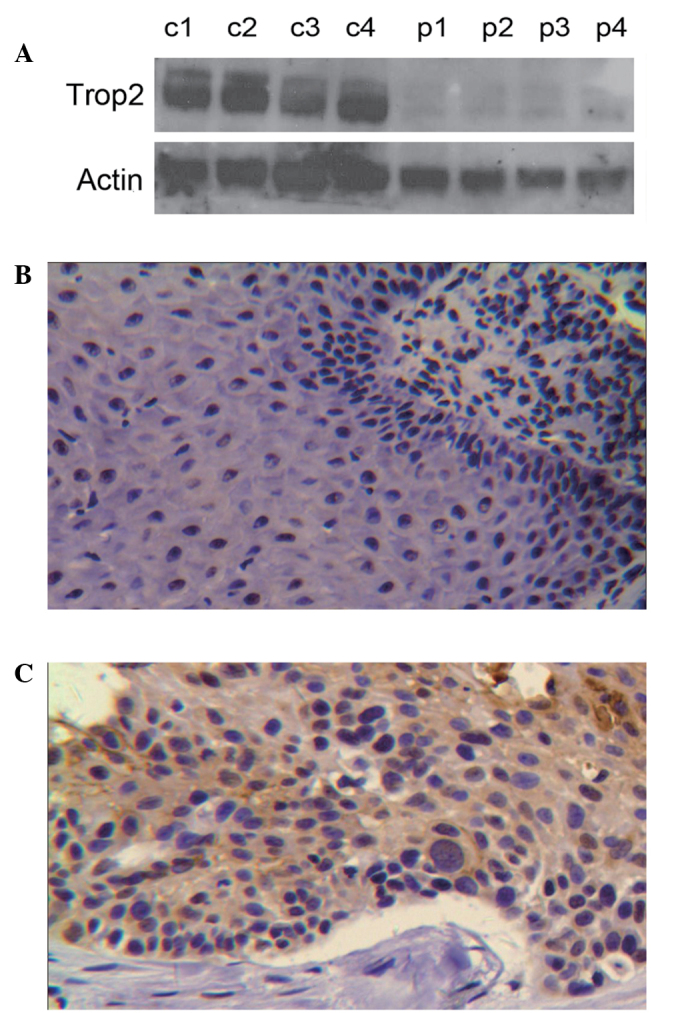
Trop2 expression in laryngeal squamous cell carcinoma tissue. (A) Western blot analysis of the protein expression of Trop2 in laryngeal carcinoma tissues (c1, c2, c3 and c4) and paired paracancerous tissues (p1, p2, p3 and p4). Actin was used as a loading control. Representative (B) negative and (C) high expression of Trop2 in paraffin embedding laryngeal carcinoma and precancerous tissues, demonstrated using immunohistochemical staining.

Trop2 protein expression levels in four fresh laryngeal carcinoma tissue samples and paracancerous tissues (control) were analyzed by western blotting (Fig. 1A). Trop2 was observed to be elevated in the carcinoma tissue compared with the control tissue. Further analysis by immunohistochemistry (IHC) demonstrated that Trop2 protein was predominantly expressed in the membrane of laryngeal carcinoma cells with a small quantity of cytoplasmic expression (Fig. 1B and C).

### Knockdown of Trop2 expression in Hep2 cells by siRNA

**Figure 2:**
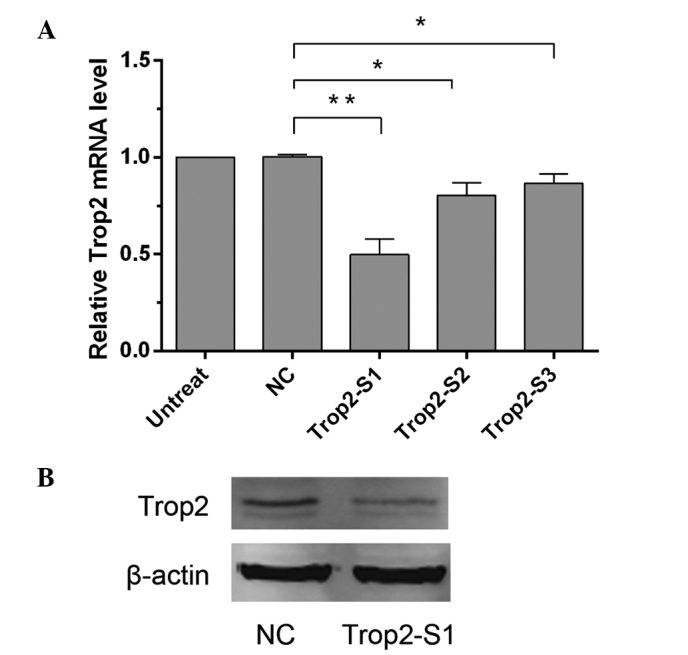
Knockdown of Trop2 expression in Hep2 cells by siRNA. (A) Trop2 mRNA expression in Hep2 cells was examined by reverse transcription-quantitative polymerase chain reaction 48 h after transfection with Trop2 siRNA or NC, as indicated (^*^P<0.05, ^**^P<0.001). (B) Western blot analysis of Trop2 protein expression in Hep2 cells 48 h following transfection with siRNA as indicated. siRNA, small interfering RNA; NC, negative control.

To investigate the role of Trop2 in LSCC, it was first silenced in Hep2 cells using siRNA transfection. Following this, cDNA generated from the Hep2 nontransfected cells, NC-transfected cells, or those transfected with three different Trop2 siRNA sequences (Trop2-S1, Trop2-S2 and Trop2-S3) was used as a template for RT-qPCR. GAPDH was used as an internal reference. The relative levels of Trop2 mRNA in the Trop2-S1, Trop2-S2 and Trop2-S3 groups were 0.50±0.18, 0.80±0.14 and 0.85±0.12, respectively, compared with the NC group (Fig. 2A). Statistical analysis revealed that, in comparison with the NC group, Trop2 expression was significantly reduced (P<0.05) following transfection with the three siRNAs, while the greatest inhibitory effect was observed with Trop2-S1 transfection. The difference between the NC (1.00±0.02) group and nontransfected group was not statistically significant (P>0.05). The Trop2-S1 group was thus selected for further assays. Similarly, as demonstrated by western blotting, the Trop2 expression in the Trop2-S1 group was significantly reduced compared with the NC group after 48 h (Fig. 2B).

### Downregulation of Trop2 inhibits viability of Hep2 cells

**Figure 3:**
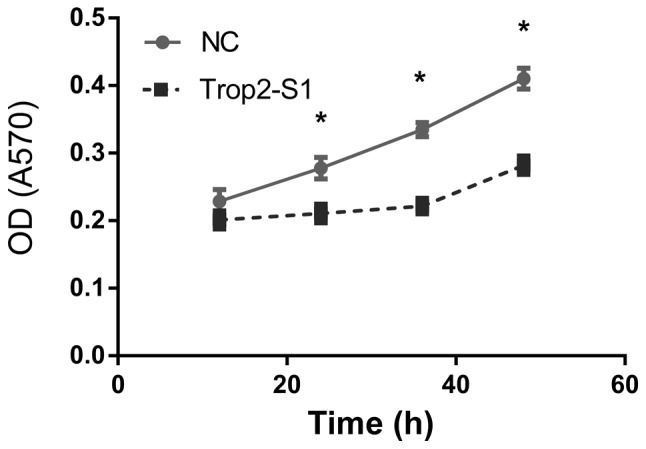
Downregulation of Trop2 in Hep2 cells inhibits viability. OD was measured at 12, 24, 36 and 48 h following transfection of Hep2 cells with control (NC) or Trop2 (Trop2-S1) using the MTT assay. At 24 h, OD was 0.21±0.02 and 0.23±0.03 in Trop2 suppressed cells and NC, respectively; OD reduced by 8.6%. At 36 h, OD was 0.24±0.03 and 0.33±0.02 in Trop2 suppressed cells and NC, respectively; OD reduced by 27.2%. At 48 h, OD was 0.29±0.04 and 0.41±0.03 in Trop2 suppressed cells and NC, respectively; OD reduced by 29.2%. ^*^P<0.05. OD, optical density; NC, negative control.

Hep2 cell viability was examined using the MTT assay at 12, 24, 36 and 48 h following Trop2 suppression. Optical density (at 570 nm) of Hep2 cells reduced following knockdown of Trop2 and the difference was statistically significant (^*^P<0.05) at 24, 36 and 48 h (Fig. 3).

### Downregulation of Trop2 inhibits cell invasion

**Figure 4:**
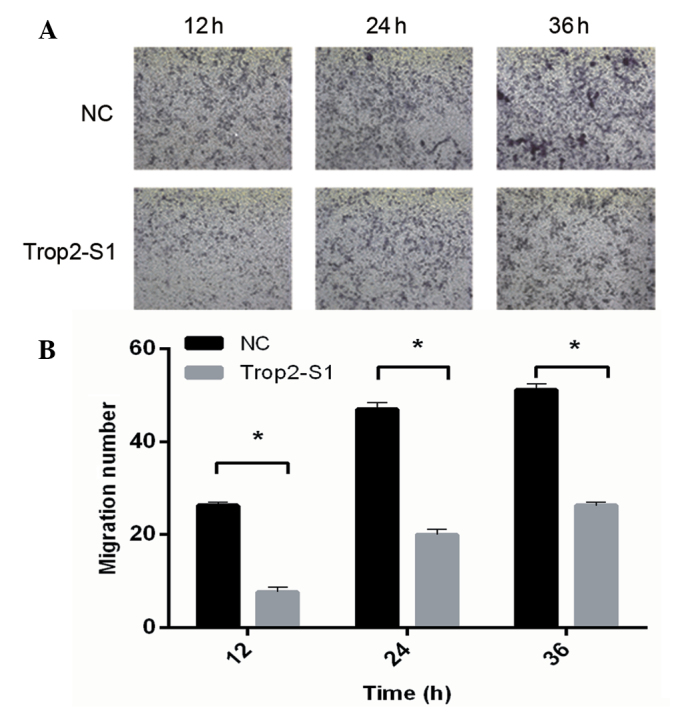
Downregulation of Trop2 inhibits cell invasion. (A) The invasive capability of Hep2 cells transfected with NC or Trop2 siRNA (Trop2-S1) was measured at the indicated time points using the Transwell assay. (B) The number of cells on the underside of the chamber was counted. NC, negative control, siRNA, small interfering RNA.

The effect of siRNA Trop2-S1 on the invasive capability of Hep2 cells was analyzed using the Transwell method. It was identified that Trop2 downregulation by Trop2-S1 was associated with the significantly reduced invasive capability of Hep2 cells (P<0.05) at all of the time-points measured. As demonstrated in Fig. 4, quantification indicated that for the Trop2-S1 and NC groups, respectively, the number of invasive cells at 12 h post-transfection was 7.02±1.26 and 23.94±0.98; at 24 h was 16.79±0.92 and 41.86±1.05; and at 36 h was 24.97±1.62 and 50.06±0.90. Thus, these data suggest that Trop2 is required for the invasive capacity of Hep2 laryngeal cells.

### Downregulation of Trop2 reduces cell migration

**Figure 5:**
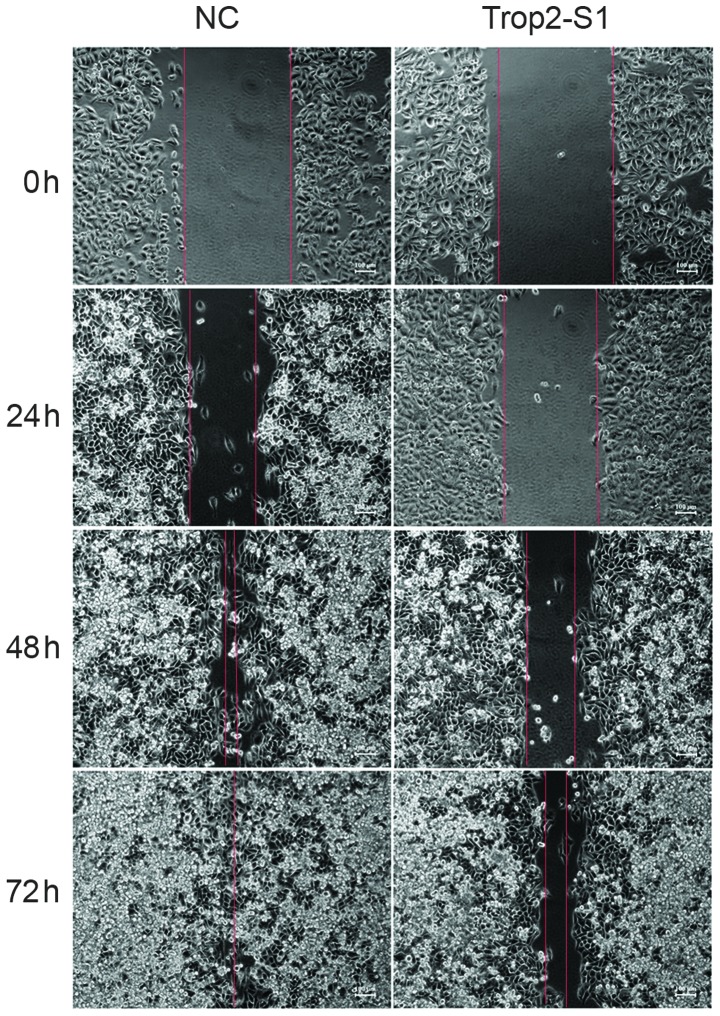
Downregulation of Trop2 inhibits cell migration. The scratch width was examined at 0, 24, 48 and 72 h. Three independent experiments were performed in which all experimental and control groups were analyzed in triplicate. Scale bar, 100 *μ*m. NC, negative control.

Next, the role of Trop2 in Hep2 cell migration was investigated. As demonstrated in the wound scratch assay, downregulation of Trop2 was associated with reduced Hep2 migration. The migratory capacity of cells transfected with Trop2-S1 was lower than that of the NC group at 24, 48 and 72 h (Fig. 5).

### MAPK/ERK signaling pathway is involved in Trop2-mediated invasion of Hep2 cells

**Figure 6:**
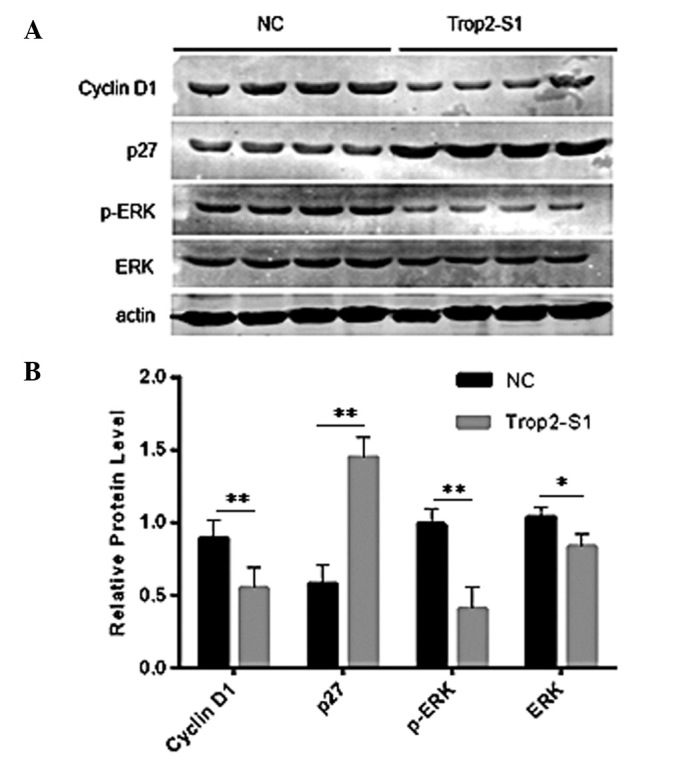
Protein expression levels of cyclin D1, p27, p-ERK and ERK following Trop2 knockdown for 48 h in Hep2 cells. (A) Western blot analysis of cyclin D1, p27, p-ERK and ERK, NC and Trop2-S1 expression levels. (B) Relative target protein expression (gray scale value of target protein/gray scale value of β-actin) was determined using Quantity One 1-D v4.62 software. ^*^P<0.05, ^**^P<0.001. ERK, extracellular signal-regulated kinase; NC, negative control.

To further evaluate the mechanism underlying Trop2 function in LSCC, the activity of the ERK/MAPK signaling pathway and associated proteins was examined in Hep2 cells with and without silencing of Trop2 by western blotting. Compared with the NC group, ERK, p-ERK and cyclin D1 expression were downregulated, while upregulation of p27 protein expression was observed in Hep2 cells following the downregulation of Trop2 (Fig. 6).

## Discussion

In a previous study, a total of 109 paraffin-embedded laryngeal carcinoma tissue specimens were analyzed using a tissue microarray for Trop2 protein expression, which was observed to be significantly increased compared with that of paracancerous tissue (13). In addition, increased Trop2 expression was observed to negatively correlate with the overall survival of patients with LSCC and was an independent prognostic factor for LSCC (13). In the current study, the Trop2 protein expression levels in fresh laryngeal carcinoma and paracancerous tissues were examined using western blotting, and Trop2 expression in carcinoma tissues was observed to be significantly increased compared with that of paracancerous tissues. The expression profile of laryngeal carcinoma tissue was also examined by IHC, which identified that Trop2 protein is predominantly expressed in the membranes of laryngeal carcinoma tissue with a small quantity of cytoplasmic expression. It has been previously demonstrated that the TP63/TP53L, ERG, GRHL1/Get-, HNF1A/TCF-1, SPI1/PU.1, WT1 and GLIS2, FOXM1 and FOXP3 transcription factor networks, which are mediated by HNF4A, can regulate the expression of Trop2 in tumor tissues (14). In addition, Trop2 has been reported to be regulated by the epigenetic regulatory factor miRNA-125b in head and neck tumors (15). A previous study demonstrated that overexpression of Trop2 is sufficient to drive cancer growth in various species (16). Upregulation of Trop-2 has been observed to quantitatively stimulate tumor growth, suggesting that it serves an oncogenic role in tumor development (16).

In the current study, Trop2 expression levels were suppressed in Hep2 laryngeal cancer cells and the resulting effects on proliferation, migration and invasiveness were examined. A total of three siRNAs (Trop2-S1, -S2 and -S3) directed against Trop2 mRNA by transfection into the Hep2 cells were screened. The results demonstrated that Trop2-S1 reduced Trop2 mRNA expression to 51% of that of the control (NC group). In addition, as validated by the western blotting assay, Trop2-S1 also reduced the expression of Trop2 protein. These results suggest that out of the three siRNAs analyzed, Trop2-S1 achieved the optimal silencing effect. Following silencing of Trop2 for 48 h, the MTT assay was conducted in order to examine Hep2 cell viability. It was observed that viability reduced 29.2% following Trop2 suppression. These results suggest that Trop2 regulates the proliferation and growth of Hep2 cells.

Previous studies have suggested that elevated Trop2 expression levels correlate with metastasis in a variety of tumor types (6,12). Thus, the migration and invasiveness of Hep2 cells were analyzed using the Transwell assay following the silencing of Trop2. The results demonstrated that the migratory and invasive abilities of Hep2 cells were reduced significantly compared with that measured in the control groups. The wound scratch assay also demonstrated that the migration of Hep2 cells with Trop2 suppression was significantly reduced. Thus, these data suggest that Trop2 regulates the invasive and migratory abilities of laryngeal carcinoma cells.

Trop2 has also been observed to regulate the activation of a number of important tumor-promoting growth factors, such as nuclear factor-κB, cyclic adenosine monophosphate response element-binding protein, Jun, retinoblastoma protein, signal transducer and activator of transcription 1 (STAT1) and STAT3, through the ERK/MAPK signaling pathway (14). In addition, Trop2 is involved in the regulation of cyclin D1 and PKC activated cell growth. Cyclin D1 is a cell cycle-dependent regulatory protein whose overexpression can result in cell growth independent of growth factors and malignant transformation of normal cells. It has been demonstrated that cyclin D1 forms a complex with cyclin-dependent kinase (CDK), which stimulates the expression of a number of downstream genes. CDK kinase may mediate this phosphorylation event and thereby the transition of cells from the G_1_ phase to S phase, in addition to the subsequent initiation of the self-division process (17).

The p27 protein is an inhibitor of cell cycle-dependent kinases and suppresses the activities of the majority of CDK-cyclin D1 complexes, subsequently inhibiting cell transition from the G_1_ phase to S phase (18). Thus, while the p27 protein maintains the quiescent state (G_0_ phase) of cells through binding to the cyclin D1-CDK complex and regulating the transition to the G_1_ phase, the ERK/MAPK signaling pathway is critical to induce cells to leave the quiescent state and initiate G_1_/S phase conversion (19).

In the current study, Trop2 expression suppression was demonstrated to result in reduced cyclin D1, ERK and p-ERK expression, together with upregulation of p27 expression and significant suppression of cell proliferation. Therefore, Trop2 is suggested to exert its suppressive effects on Hep2 cell proliferation through the suppression of ERK expression and phosphorylation, and subsequent downregulation of cyclin D1 expression and upregulation of p27 expression.

In conclusion, as demonstrated by the current study, Trop2 is suggested to regulate the growth, invasion and migration of laryngeal carcinoma cells through the ERK/MAPK signaling pathway. Therefore, although further studies are required, including validation in animal studies, Trop2 is suggested as a novel target for molecular therapy against laryngeal carcinoma.

## Figures and Tables

**Table I:** Candidate siRNA sequences of Trop2.

Name	Sequences (5′–3′)	Position in Trop2 mRNA (bp)
Trop2-S1	GUGUCCCACCAACAAGAUGTT	443
Trop2-S2	CCAAGUGUCUGCUGCUCAATT	550
Trop2-S3	GCACGCUCAUCUAUUACCUTT	1100
Trop2-NC	UUCUCCGAACGUGUCACGUTT	

siRNA, small interfering RNA; bp, base pairs; NC, negative control.

**Table II:** Primers for Trop2 mRNA detection by reverse transcription-quantitative polymerase chain reaction.

Name	Primers
Trop2	Fwd: 5′-TATTACCTGGACGAGATTCCCC-3′
	Rev: 5′-CCCCGACTTTCTCCGGTTG-3′
GAPDH	Fwd: 5′-TGCACCACCAACTGCTTAGC-3′
	Rev: 5′-GGCATGGACTGTGGTCATGAG-3′
